# Risk stratification of smoldering multiple myeloma incorporating revised IMWG diagnostic criteria

**DOI:** 10.1038/s41408-018-0077-4

**Published:** 2018-06-12

**Authors:** Arjun Lakshman, S. Vincent Rajkumar, Francis K. Buadi, Moritz Binder, Morie A. Gertz, Martha Q. Lacy, Angela Dispenzieri, David Dingli, Amie L. Fonder, Suzanne R. Hayman, Miriam A. Hobbs, Wilson I. Gonsalves, Yi Lisa Hwa, Prashant Kapoor, Nelson Leung, Ronald S. Go, Yi Lin, Taxiarchis V. Kourelis, Rahma Warsame, John A. Lust, Stephen J. Russell, Steven R. Zeldenrust, Robert A. Kyle, Shaji K. Kumar

**Affiliations:** Division of Hematology, Mayo Clinic, Rochester, MN USA

## Abstract

In 2014, the International Myeloma Working Group reclassified patients with smoldering multiple myeloma (SMM) and bone marrow-plasma cell percentage (BMPC%) ≥ 60%, or serum free light chain ratio (FLCr) ≥ 100 or >1 focal lesion on magnetic resonance imaging as multiple myeloma (MM). Predictors of progression in patients currently classified as SMM are not known. We identified 421 patients with SMM, diagnosed between 2003 and 2015. The median time to progression (TTP) was 57 months (CI, 45–72). BMPC% > 20% [hazard ratio (HR): 2.28 (CI, 1.63–3.20); *p* < 0.0001]; M-protein > 2g/dL [HR: 1.56 (CI, 1.11–2.20); *p* = 0.01], and FLCr > 20 [HR: 2.13 (CI, 1.55–2.93); *p* < 0.0001] independently predicted shorter TTP in multivariate analysis. Age and immunoparesis were not significant. We stratified patients into three groups: low risk (none of the three risk factors; *n* = 143); intermediate risk (one of the three risk factors; *n* = 121); and high risk (≥2 of the three risk factors; *n* = 153). The median TTP for low-, intermediate-, and high-risk groups were 110, 68, and 29 months, respectively (*p* < 0.0001). BMPC% > 20%, M-protein > 2 g/dL, and FLCr > 20 at diagnosis can be used to risk stratify patients with SMM. Patients with high-risk SMM need close follow-up and are candidates for clinical trials aiming to prevent progression.

## Introduction

The term smoldering multiple myeloma (SMM) was first introduced in 1980 to identify a group of six patients with 10% or more plasma cells (PCs) in the bone marrow (BM) and no organ damage at diagnosis, who did not develop organ dysfunction related to multiple myeloma (MM) for more than 5 years^1^. Since then, the designation has been expanded to include patients who have ≥10% clonal PCs in the BM, ≥3 g/dL of monoclonal protein (M-protein) in serum (or ≥500 mg/24 h in urine) or both, in the absence of any end-organ damage^2^. SMM is a clinically defined, heterogeneous entity, and includes patients with a pre-clinical malignancy who progress to active end-organ damage, as well as those with a pre-malignant state with a low rate of progression to MM or other lymphoproliferative disorders. Several classification systems have been developed to identify patients with SMM at a higher risk of progression who require aggressive monitoring, and to identify candidates for investigational therapies to reduce risk of progression. The Mayo Clinic and the Spanish models were used to identify patients with high-risk SMM in a clinical trial of lenalidomide and dexamethasone in high-risk SMM^3^. The Mayo Clinic model uses M-protein (≥3 g/dL), BMPC% (≥10%), and the ratio of involved to uninvolved serum free light chains (FLCr) (≥8) to categorize patients into three risk categories, with a 76% risk of progression in 5 years among those with all three of the above characteristics^4,5^. The Spanish model uses the proportion of BMPCs with aberrant PC phenotype on flow cytometry (≥95%) and reduction in uninvolved immunoglobulins (immunoparesis) to identify high-risk patients^6^. Abnormalities detected on imaging of spine or whole body using magnetic resonance imaging (MRI), and underlying cytogenetic abnormalities also guide clinicians in identifying high-risk patients^7–13^.

The previous diagnostic criteria for MM required evidence of organ damage (in the form of CRAB features—hypercalcemia, renal dysfunction, anemia, and bone lesions) attributable to clonal PC proliferation to diagnose MM and institute treatment^2^. Since then, several studies showed that BMPC% ≥ 60%, presence of >1 focal lesion on MRI, or FLCr ≥ 100 at diagnosis of SMM (biomarkers) were associated with an approximately 80% risk of progression at 2 years^9,11,14–16^. Since much of the morbidity and mortality in MM are related to the organ damage, the 2014 revision of the diagnostic criteria for PC disorders by the International Myeloma Working Group (IMWG) reclassified these “ultra-high-risk” patients with SMM (~10% of SMM) as MM requiring therapy^17^. With the advent of the new definition, the risk factors for progression and best cutoffs for disease markers for defining risk of progression in the remaining patients with SMM are not known. Besides, with the wider availability of more sensitive imaging modalities such as MRI, whole-body low-dose computed tomography (CT), and positron emission tomography with CT (PET-CT) scan, skeletal lesions can be detected earlier compared to the standard radiological skeletal survey^18,19^. In this context, we examined a cohort of patients with SMM who met the 2014 IMWG criteria and were seen at our institution between 2003 and 2015 to define their natural history and identify the risk factors for progression.

## Patients and methods

### Patients

We reviewed the Dysproteinemia database at Mayo Clinic, Rochester, to identify patients who had a diagnosis of SMM made between 2003 and 2015. All patients had BMPC% ≥ 10% and/or serum M-protein level ≥ 3 g/dL (or ≥500 mg/24 h in urine), and no CRAB features related to the PC proliferative disorder. We excluded the following patients, so that our cohort closely aligned with the patients who satisfy the current diagnostic criteria for SMM^1^: those with BMPC% ≥ 60%, and/or FLCr ≥ 100 and involved FLC level > 10 mg/dL, and/or those who underwent an MRI examination at diagnosis and had >1 focal lesion (*n* = 88)^2^, and when the exact BMPC% (*n* = 17), FLC levels (*n* = 260), or both (*n* = 2) were not available at diagnosis^17^. We reviewed the electronic medical records to abstract data regarding demographics, laboratory tests at diagnosis, availability of an advanced imaging of the axial skeleton at diagnosis (MRI, PET-CT, or CT scan), timing of progression of SMM, and survival status at last follow-up. All patients underwent a radiological skeletal survey at diagnosis. We defined advanced imaging as PET-CT scan, whole-body MRI, or MRI whole spine with or without pelvis performed within 3 months from the diagnosis of SMM. We defined immunoparesis as reduction in one or more of the uninvolved immunoglobulins below the lower limit of normal^4^. The data cutoff date was 30 June 2017. The Mayo Clinic Institutional Review Board approved the study. The study was conducted in accordance with the Declaration of Helsinki and the Health Insurance Portability and Accountability Act guidelines of 1996.

### Outcome measures

Progression of SMM was defined as development of organ damage attributable to PC dyscrasia (one or more of CRAB features defined using cutoffs as proposed in the 2014 IMWG criteria for diagnosis of MM), initiation of therapy for MM in the absence of CRAB features, or development of immunoglobulin light chain amyloidosis^17^. Time to progression (TTP) was calculated as the duration from diagnosis of SMM to the date of starting therapy. Patients were censored in the TTP analysis if they did not progress at the date of last follow-up, started therapy with an anti-myeloma agent on a clinical trial for SMM, or initiated therapy with systemic corticosteroid or anticancer chemotherapy for any other indication. Patients who had not progressed at the time of death were also censored. Overall survival (OS) was defined as the duration from diagnosis of SMM to the date of death or last follow-up, patients being censored if they were alive.

### Interphase fluorescent in situ hybridization

We abstracted the results of first available fluorescent in situ hybridization (FISH) test before disease progression. BM aspirate samples enriched for mononuclear cells by the Ficoll method were used for preparing cytospin slides. FISH analysis was performed in conjunction with cytoplasmic immunoglobulin staining (cIg-FISH) as described previously using the following probes: 3cen (D3Z1); 7cen (D7Z1); 9cen (D9Z1); 15cen (D15Z4); 11q13 (CCND1-XT); 14q32 (IGH-XT); 13q14 (RB1); 13q34 (LAMP1); 17p13.1 (p53); 17cen (D17Z1); 4p16.3 (FGFR3); 16q23 (c-MAF); 6p21 (CCND3); and 20q12 (MAFB)^20^.

### Statistical analysis

We summarized categorical variables as proportions and continuous variables as medians (range). We performed receiver operating characteristic (ROC) curve with area under the curve (AUC) analysis to define optimal cutoffs for BMPC%, M-protein, and FLCr, using progression within 3 years from diagnosis as a binary end point. For this calculation, we considered all patients who had not progressed at 3 years as non-progressors (*n* = 154), and those who progressed within 3 years as progressors (*n* = 114). In this calculation, we excluded patients who were censored within 3 years in the TTP analysis (*n* = 153). We performed time-to-event analyses using the Kaplan–Meier method. We used Cox proportional hazards model to elucidate the impact of putative predictors on TTP. All variables with a *p*-value < 0.1 in univariate analysis were included in the multivariate model, and we used backward selection to arrive at the final model. ROC analysis was used to determine cutoffs and performance characteristics of the derived scoring system. The equality of the ROC curves of the conventional and the new risk stratification system were compared using a non-parametric approach^21^. A two-sided *p*-value < 0.05 was considered significant for all statistical tests. We performed statistical analysis using the JMP^®^ Pro 12.0 software package (SAS Institute Inc., Cary, NC, USA) and the Stata software (version 15.1, StataCorp, College Station, USA).

## Results

### Patient characteristics

We identified 421 patients who satisfied the inclusion criteria. Their baseline demographic and laboratory characteristics are shown in Table 1. Eighteen (4.5%) patients had a hemoglobin value at diagnosis of <10 g/dL, the causes being anemia of chronic kidney disease (*n* = 6), myelodysplasia (*n* = 4), anemia of chronic disease (*n* = 4), nutritional deficiency (*n* = 3), and thalassemia minor (*n* = 1). Two (0.5%) patients had a serum calcium value > 11 mg/dL, attributed to sarcoidosis (*n* = 1) and primary hyperparathyroidism (*n* = 1) and it was corrected with treatment. A serum creatinine > 2 mg/dL in 11 (2.8%) patients was attributed to unrelated chronic kidney disease (*n* = 10) and analgesic-induced acute kidney injury (*n* = 1). FLCr was ≥100 in 6 (1.4%) patients, but none of them had an involved FLC level ≥ 10 mg/dL.

**Table 1:** Patient characteristics at diagnosis of smoldering multiple myeloma (*n* = 421)

Age, years, median (range)	64.9 (30.2–92.1)
Gender, *n* (%)	
• Female	176 (41.8)
• Male	246 (58.2)
Hemoglobin (*n* = 403), g/dL, median (range)	12.7 (7.8–17.3)
Serum calcium (*n* = 391), g/dL, median (range)	9.4 (7.8–15.3)
Serum creatinine (*n* = 398), mg/dL, median (range)	1 (0.2–5.5)
Serum M-protein (*n* = 417), median (range)	2 (0–5.0)
• M-protein > 2 g/dL, *n* (%)	195 (46.8)
BMPC percentage, median (range)	20 (5–50)
• BMPC percentage > 20, *n* (%)	142 (33.7)
Involved to uninvolved free light chain ratio, median (range)	7.8 (0.3–281.5)
• FLCr > 20, *n* (%)	125 (29.7)
Heavy chain isotype, *n* (%)	
• IgG	319 (75.8)
• IgA	83 (19.7)
• IgM	4 (0.9)
• Light chain only and others	15 (3.6)
Immunoparesis^a^ (*n* = 372), *n* (%)	262 (70.4)
LDH ≥ upper limit of normal (*n* = 303), *n* (%)	26 (8.6)
Serum albumin < 3.5 g/dL (*n* = 386), *n* (%)	119 (30.8)
Serum beta-2-microglobulin ≥ 3.5 mg/dL (*n* = 347), *n* (%)	89 (25.6)
Advanced imaging at or within 3 months from diagnosis, *n* (%)	124 (29.5)
• Whole-body PET-CT	110 (88.7)
• MRI whole spine with pelvis	8 (6.5)
• MRI whole spine without pelvis	6 (4.8)
Bone marrow-plasma cell FISH, *n* (%)	297 (70.5)
• t(4;14)	33 (11.1)
• t(11;14)	47 (15.8)
• t(14;16)	7 (2.4)
• t(6;14)	2 (0.7)
• t(14;20)	2 (0.7)
• IgH translocation with unknown partner/deletion	43 (14.5)
• Hyperdiploidy	129 (43.4)
• Monosomy 13/del(13q)	89 (30.0)
• Del(17p)	7 (2.4)
• Normal FISH/ none of the above abnormalities^b^	60 (20.2)
• Insufficient plasma cells in BM aspirate	5 (1.7)

*BMPC* bone marrow-plasma cell, *FLCr* ratio of involved to uninvolved serum free light chain, *FISH* interphase fluorescent in situ hybridization, *LDH* lactate dehydrogenase, *MRI* magnetic resonance imaging, *PET-CT* positron emission tomography with computed tomography

^a^ For definition of immunoparesis, the lower limits of normal for immunoglobulins were as follows: IgG—600 mg/dL; IgA—50 mg/dL; and IgM—50 mg/dL

^b^ We did not consider presence of del(1p) and gain(1q) for this calculation

### Outcomes

Over an estimated median follow-up period of 74.8 months (95% confidence interval (CI), 67.7–83.0), progression was documented in 165 (39.2%) patients; 158 of them developed MM while 7 developed AL amyloidosis. The estimated median TTP for the entire cohort was 57.3 months (95% CI, 44.8–72.2). The estimated proportion of patients progressing at 2, 5, and 10 years were 28.8% (95% CI, 24.1–34.0), 51.0% (95% CI, 44.8–57.2), and 71.2% (95% CI, 60.8–79.8), respectively. The risk of progression was 18% during the first year, approximately 10% per year for the next 3 years, and about 4% per year thereafter till 10 years from diagnosis. Among patients who developed MM, the most common myeloma defining events were anemia (*n* = 65; 41%) and skeletal lesions (*n* = 53; 33%). Renal dysfunction was seen in 7 (4%) and hypercalcemia in 3 (2%). Forty-five (28.4%) patients were treated in the absence of CRAB features due to markers like high M-protein (>5 g/dL), high BMPC%, and rapidly rising FLC level. During follow-up, 127 (30.2%) patients died. The median OS for the entire cohort was 11.3 years (95% CI, 9.8–not reached).

### Risk factors for progression

The putative risk factors we initially considered for inclusion in the univariate analysis were gender, BMPC%, M-protein level, FLCr, M-protein isotype (IgG vs. non-IgG and IgA vs. non-IgA), and presence of immunoparesis. We used progression at 3 years as a binary end point to perform ROC curve analysis to define optimal cutoffs for BMPC%, M-protein, and FLCr as described earlier. We obtained BMPC% of 20% (sensitivity—72%; specificity—67%; AUC—0.73), M-protein of 2.1 g/dL (sensitivity—62%; specificity—60%; AUC—0.62), and FLCr of 18.8 (sensitivity—52%; specificity—76%; AUC—0.64) as best cutoffs. For convenience, we decided to use BMPC% > 20% vs. ≤20%, M-protein > 2 g/dL vs. ≤2 g/dL, and FLCr > 20 vs. ≤20 for stratifying the patients in the analysis. In univariate analysis, BMPC% > 20%, M-protein > 2 g/dL, FLCr > 20, and presence of immunoparesis were associated with shorter TTP (Table 2).

**Table 2:** Univariate and multivariable analysis for risk factors for progression in smoldering multiple myeloma

Risk factor	Time to progression, months, median (95% CI)	Univariate model^a^		Multivariable model^a,b^	
		Hazard ratio (95% CI)	*p*	Hazard ratio (95% CI)	*p*
Gender					
• Male (*n* = 245)	55.0 (41.0–109.8)	0.95 (0.70–1.29)	0.735	NI	—
• Female (*n* = 176)	57.3 (43.3–73.2)				
BMPC percentage					
• >20% (*n* = 142)	29.8 (15.9–35.9)	2.79 (2.05–3.81)	**<0.0001**	2.28 (1.63–3.20)	**<0.0001**
• ≤20% (*n* = 279)	83.1 (64.9–126.9)				
Serum M-protein					
• >2 g/dL (*n* = 195)	38.3 (29.8–44.4)	2.07 (1.51–2.85)	**<0.0001**	1.56 (1.11–2.20)	**0.010**
• ≤2 g/dL (*n* = 222)	109.8 (63.0–NR)				
FLCr					
• >20 (*n* = 125)	30.8 (19.8–40.6)	2.23 (1.63–3.04)	**<0.0001**	2.13 (1.55–2.93)	**<0.0001**
• ≤20 (*n* = 296)	83.1 (63.0–109.8)				
Immunoparesis					
• Present (*n* = 262)	50.6 (40.6–67.8)	1.59 (1.07–2.45)	**0.022**	1.01 (0.66–1.60)	0.957
• Absent (*n* = 110)	109.8 (58.1–NR)				
M-protein isotype					
• IgG (*n* = 319)	57.3 (43.5–72.2)	0.94 (0.67–1.37)	0.761	NI	—
• Non-IgG (*n* = 102)	62.1 (35.0–105.0)				
• IgA (*n* = 83)	65.1 (35.0–NR)	1.04 (0.70–1.51)	0.823	NI	—
• Non-IgA (*n* = 338)	55.0 (43.4–69.1)				

Values in bold indicate statistically significant *p*-values

Abbreviations as explained in Table 1; *NI* not included in the analysis, *NR* not reached

^a^ Cox proportional hazards model

^b^ *N* = 370 for the full model for multivariable analysis incorporating age, BMPC%, serum M-protein, FLCr, immunoparesis, and M-protein isotype; *N* = 417 for the final model after backward selection incorporating BMPC%, serum M-protein, and FLCr

**Fig. 1:**
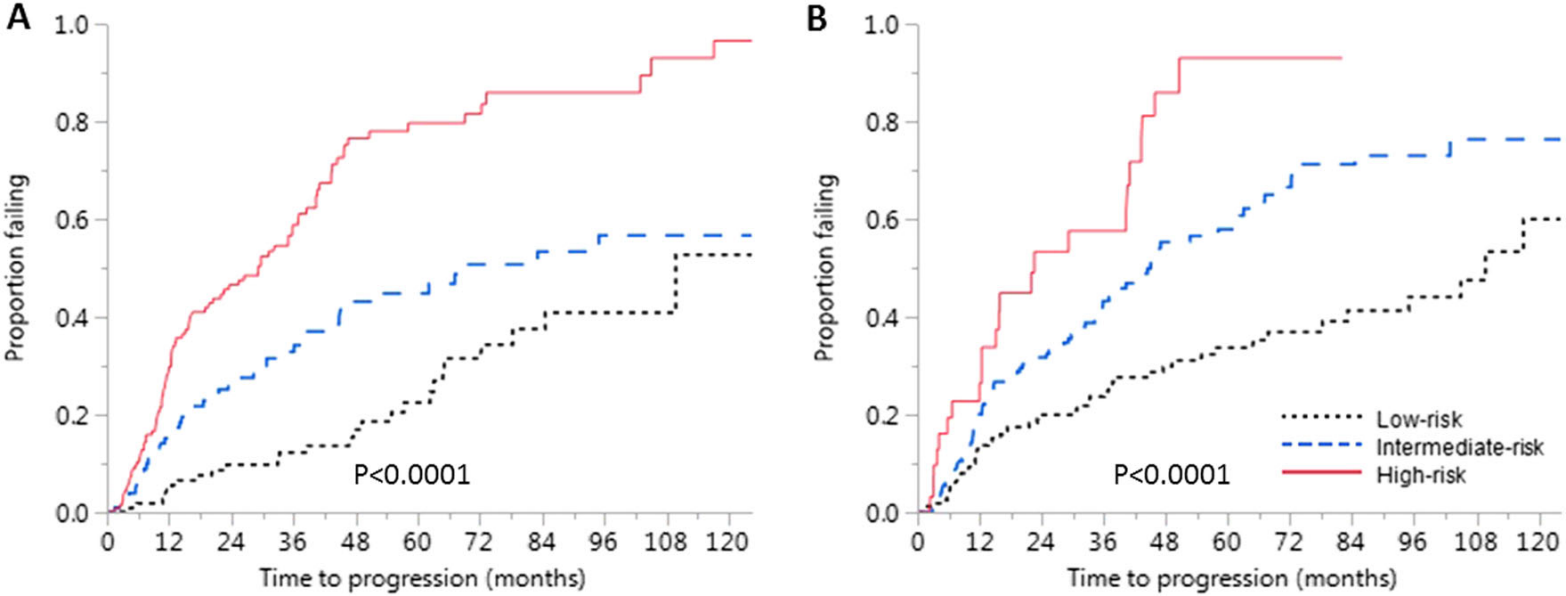
Time to progression in the three risk categories using the proposed and conventional Mayo Clinic models. **a** Kaplan–Meier failure curves showing time to progression (TTP) in patients with none (low risk), one (intermediate risk), and two or more (high risk) of bone marrow-plasma cell percentage (BMPC%) > 20%, monoclonal protein > 2 g/dl, and free light chain ratio (FLCr) > 20 at diagnosis of smoldering multiple myeloma. The estimated median TTP in the low-risk, intermediate-risk, and high-risk groups were 109.8 months (95% CI, 78.3–not reached), 67.8 months (95% CI, 44.8–not reached), and 29.2 months (95% CI, 16.5–36.9), respectively (*p* < 0.0001). **b** Kaplan–Meier failure curves for patients stratified according to the conventional Mayo Clinic model by presence of one (low risk), two (intermediate risk), and three (high risk) of monoclonal protein ≥ 3 g/dL, BMPC% ≥ 10%, and FLCr ≥ 8 at diagnosis. The estimated median TTP in the three groups were 109.8 months (95% CI, 83.1–126.9), 45.1 months (95% CI, 35.8–62.1), and 22.6 months (95% CI, 12.4–41.0), respectively (*p* < 0.0001). The proposed classification system performed better than the conventional system by area under the curve analysis

We included BMPC%, M-protein, FLCr, and immunoparesis in the multivariable analysis. BMPC%, M-spike, and FLCr were associated with shorter TTP in the multivariable model (Table 2). We then proceeded to construct a risk stratification system. Of the 417 patients who had all the three variables available, 143 (34.3%) had none of the three risk factors, 121 (29%) had one of the three risk factors, and 120 (28.8%) patients had two out of the three risk factors. Thirty-three (7.9%) patients had all three risk factors. We assigned the patients with none of the risk factors to the “low-risk” category (*n* = 143), those with one risk factor to the “intermediate-risk” category (*n* = 121), and patients with two or more risk factors to the “high-risk” category (*n* = 153; 36.7%), given that there was no significant difference in median TTP between patients with two or three risk factors. The estimated median TTP in the low-risk, intermediate-risk, and high-risk groups were 109.8 months (95% CI, 78.3–not reached), 67.8 months (95% CI, 44.8–not reached), and 29.2 months (95% CI, 16.5–36.9), respectively (*p* < 0.0001; Fig. 1a). The hazard ratio (HR) for progression in the high-risk group with respect to the low-risk and intermediate-risk groups were 5.1 (95% CI, 3.37–8.06) and 2.53 (95% CI, 1.77–3.69), respectively. The estimated risk of progression at 2, 5, and 10 years from diagnosis for the three groups and the odds ratios for progression relative to the low-risk group in the intermediate and high-risk groups are given in Table 3. The low-risk group had a 5% per year risk of progression during the first 10 years. The rates of progression in the intermediate-risk group were approximately 15% per year during the first 2 years, 7% per year during the next 3 years, and 4% per year thereafter up to 10 years. In the high-risk group, the risk of progression was 24% per year during the first 2 years; it then decreased to 11% per year for the next 3 years and then to 3% per year up to 10 years. For patients who had all three risk factors, the estimated rates of progression at 2 and 5 years were 64.7% and 92.6%, respectively. We excluded 61 (14.5%) patients who received therapy for SMM on a clinical trial, or received corticosteroid or anticancer therapy for other indications, and repeated the analysis and the results were consistent.

**Table 3:** Estimated rate of progression and odds ratio for progression in patients with smoldering multiple myeloma in low-, intermediate-, and high-risk groups using BMPC% > 20%, M-protein > 2 g/dL, and FLCr > 20

Time from diagnosis (years)	Low risk (*n* = 143)	Intermediate risk (*n* = 121)		High risk (*n* = 153)	
	Estimated rate of progression (%)	Rate of progression, % (CI)	OR for progression relative to low-risk group (CI)	Rate of progression, % (CI)	OR for progression relative to low-risk group (CI)
2	9.7 (5.3–17.1)	26.3 (18.4–36.2)	2.71 (1.08–6.83)	47.4 (38.6–56.4)	4.89 (2.25–10.69)
5	22.5 (14.2–33.6)	46.7 (35.8–57.9)	2.08 (1.07–4.08)	81.5 (71.3–88.6)	3.63 (2.12–6.22)
10	52.7 (30.1–74.2)	65.3 (45.5–80.9)	1.24 (0.61–2.69)	96.5 (80.9–99.4)	1.83 (1.09–3.30)

*BMPC%* bone marrow-plasma cell percentage, *CI* 95% confidence intervals, *FLCr* involved to uninvolved free light chain ratio, *OR* odds ratio

We applied the conventional Mayo Clinic model for identifying low-, intermediate-, and high-risk patients to our study population (Fig. 1b). The median TTP for the low-, intermediate-, and high-risk groups as defined by the conventional model were 109.8 months (95% CI, 83.1–126.9), 45.1 months (95% CI, 35.8–62.1), and 22.6 months (95% CI, 12.4–41.0), respectively. We then compared our proposed model using the new cutoffs with the conventional Mayo Clinic model using progression at 2, 3, and 5 years as end points, and our new model consistently performed better. The AUCs for our proposed classification and the conventional Mayo clinic classification were 0.71 (95% CI, 0.65–0.76) and 0.62 (95% CI, 0.55–0.68; *p* = 0.004) at 2 years; 0.73 (95% CI, 0.67–0.79) and 0.62 (95% CI, 0.56–0.68; *p* = 0.0004) at 3 years; and 0.77 (95% CI, 0.71–0.83) and 0.68 (95% CI, 0.62–0.75; *p* = 0.010) at 5 years, respectively (Supplementary appendix). Table 4 shows the distribution of patients using the conventional Mayo Clinic model and their reclassification according to the proposed risk stratification. A detailed categorization of patients incorporating each risk factor in each risk category for the two systems is given in Supplementary appendix.

**Table 4:**
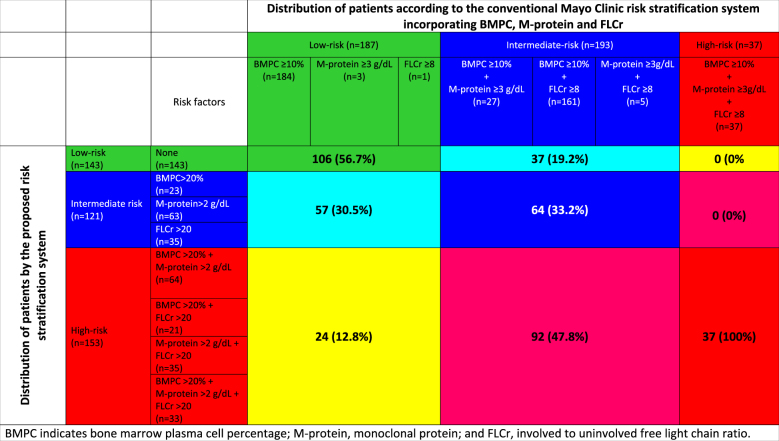
Comparison of risk stratification of patients using the conventional Mayo Clinic model and the proposed classification system

*BMPC* bone marrow-plasma cell percentage, *M-protein* monoclonal protein, *FLCr* involved to uninvolved free light chain ratio

In a subgroup analysis, we applied the model specifically to patients who had any advanced imaging at diagnosis (*n* = 124). The median TTP in the low-, intermediate-, and high-risk groups were not reached (95% CI, 63.0 months–not reached), not reached (95% CI, 14.6 months–not reached), and 15.7 months (95% CI, 12.0–29.8), respectively (*p* < 0.0001). The estimated 2- and 5-year progression rates for the above groups were 7 and 31%, 36 and 46%, and 60 and 100% respectively. Interestingly, patients who had an advanced imaging at diagnosis showed a trend toward shorter TTP when compared to those who did not have an advanced imaging: 43.4 months (95% CI, 29.8–69.1) vs. 62.4 months (95% CI, 45.9–83.1; *p* = 0.059).

Given the prognostic value of cytogenetic abnormalities in SMM, we then tested the significance of the three risk factors in the subset of patients who had undergone FISH testing before progression (*n* = 297). Among these, 156 (52.5%) patients had t(4;14) and/or del(17p) and/or hyperdiploidy (designated high risk as they have been associated with higher risk of progression)^12,13^. We included presence vs. absence of high-risk abnormalities along with the three risk factors previously identified in the entire cohort in the multivariate model. BMPC% > 20% [HR: 2.43 (95% CI, 1.68–3.52); *p* < 0.0001], FLCr > 20 [HR: 2.79 (95% CI, 1.91–4.07); *p* < 0.0001], and high-risk cytogenetics [HR: 1.70 (95% CI, 1.17–2.51); *p* = 0.005] were associated with higher risk of progression while M-protein > 2 g/dL was not [HR: 1.41 (95% CI, 0.95–2.14); *p* = 0.090]. The median TTP in patients with none, 1, or 2 or more of the above risk factors were not reached (95% CI, 72.4 months–not reached), 83.1 months (95% CI, 52.7–124.8), and 23.6 months (95% CI, 14. 6–36.1), respectively (*p* < 0.0001). The estimated rates of progression at 5 years in the above groups were 20%, 38%, and 81% respectively. The 2-year progression rate with two or three of the above risk factors was 50%.

**Fig. 2:**
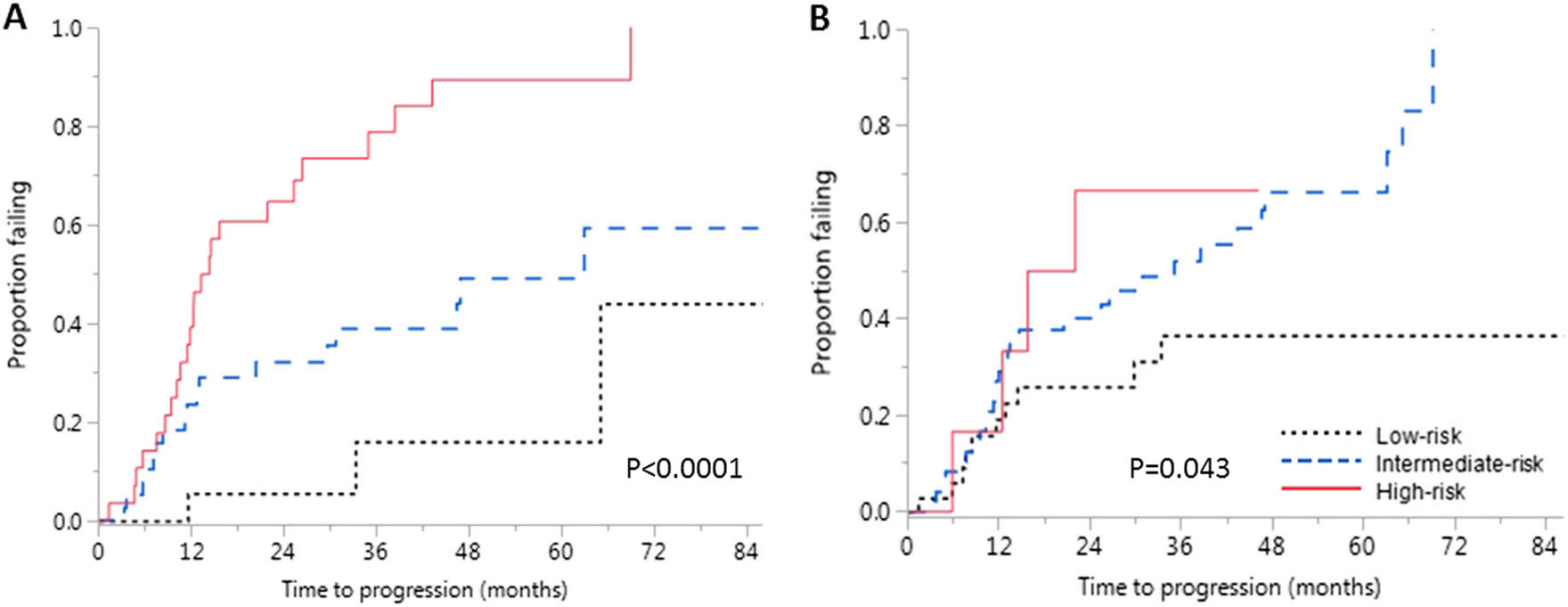
Risk stratification of smoldering multiple myeloma in a subset of patients with FISH testing and advanced imaging available at diagnosis. **a** Kaplan–Meier failure curves showing time to progression (TTP) in patients with none (low risk), one (intermediate risk), and two or more (high risk) of bone marrow-plasma cell percentage (BMPC%) > 20%, free light chain ratio (FLCr) > 20, and high-risk cytogenetics [del17p, t(4;14) or hyperdiploidy]. The estimated median TTP in the three groups were not reached (95% CI, 33.3 months–NR), 63.0 months (95% CI, 29.8–NR), and 14.5 months (95% CI, 10.7–25.4), respectively (*p* < 0.0001). **b** Kaplan–Meier failure curves for patients stratified according to the conventional Mayo Clinic model by presence of one (low risk), two (intermediate risk), and three (high risk) of monoclonal protein ≥ 3 g/dL, BMPC% ≥ 10%, and FLCr ≥ 8 at diagnosis. The estimated median TTP in the three groups were NR (95% CI, 29.8 months–NR), 35.1 months (95% CI, 13.4–47.0), and 18.9 months (95% CI, 5.8–NR), respectively (*p* = 0.043)

When we applied the above three risk factors (BMPC, FLCr, and high-risk cytogenetics) to patients with an advanced imaging and FISH available (*n* = 102), the median TTP in those with none (*n* = 26), one (*n* = 46), and two or three (*n* = 30) risk factors were not reached (95% CI, 33.3 months–NR), 63.0 months (95% CI, 29.8–NR), and 14.5 months (95% CI, 10.7–25.4), respectively (*p* < 0.0001; Fig. 2). The estimated 2- and 5-year progression rates in the three groups were 6 and 16%, 32 and 59%, and 69 and 100%, respectively. In this subgroup, the AUC for a combination of BMPC, FLCr, and high-risk cytogenetics for predicting progression at 2, 3, and 5 years were numerically better than the conventional Mayo Clinic Model, even though we could not demonstrate a statistically significant improvement in predictability across all time points considering the small number of patients. The AUC for our new model incorporating cytogenetics and the conventional Mayo Clinic model for predicting progression were 0.74 (95% CI, 0.64–0.84) and 0.60 (95% CI, 0.49–0.73; *p* = 0.019) at 2 years, 0.75 (95% CI, 0.64–0.86) and 0.58 (95% CI, 0.45–0.70; *p* = 0.004) at 3 years, and 0.80 (95% CI, 0.65–0.94) and 0.70 (95% CI, 0.54–0.86; *p* = 0.232) at 5 years, respectively (Supplementary appendix).

## Discussion

Our study redefines the cutoffs for markers at diagnosis for risk stratification of SMM. We propose a simple risk stratification system based on BMPC%, M-protein level, and FLCr, their cutoffs being different from the conventional Mayo Clinic model^4,5^. Presence of two or more of these risk factors defines a population at a distinctly higher risk of progression compared to other patients. BMPC% is a direct indicator of clonal PC burden, while M-protein and FLCr are surrogate markers for clonal PC expansion^22,23^. The principal advantage of our model is that it uses widely available and routinely performed tests to risk stratify patients at diagnosis. Our new model performed better than the conventional Mayo Clinic model in risk stratifying patients.

Multiple series have examined the TTP and factors, which predict progression in patients with asymptomatic MM and the results vary depending upon the diagnostic criteria used to define the patient cohort. Earlier studies included patients who had less than three lytic bone lesions, but did not have symptoms related to them, and showed that presence of lytic lesions, M-protein level ≥ 3 g/dL, progressive rise in the M-protein during follow-up, IgA subtype, and urinary excretion of Bence Jones protein (>200 mg/day) were predictors of progression^24–28^. Subsequent studies excluded patients with bone lesions. M-protein level, IgA subtype, and Bence Jones protein excretion (>50 mg/day) were identified as risk factors in one study^8^. BMPC% > 10%, IgA subtype, and detectable Bence Jones proteinuria predicted progression in another study^29^. The Mayo Clinic group examined a group of 276 patients with SMM diagnosed between 1970 and 1995, with median follow-up of over 10 years and reported that the median TTP in the cohort was 4.8 years (approximately 58 months). BMPC% ≥ 10%, M-protein level ≥ 3 g/dL, and FLCr ≥ 8 were identified as independent predictors of TTP at diagnosis, and presence of one, two, or three of these factors were used to define three risk groups with differing rates of progression^4,5^. The above cohort included patients with BMPC% ≥ 60% (*n* = 6) and patients with FLCr ≥ 100^4,5,14^. The median TTP of 57 months seen in our cohort is comparable to that of the above cohort; but considering that the higher-risk patients were excluded, we expected the TTP to be longer. This mismatch can be explained by^1^ a proportion of patients in our study were treated for MM before developing CRAB features because their treating physicians thought that they were at high risk of complications by virtue of high M-protein level or FLC level; and^2^ many patients who developed skeletal lesions in the current cohort were diagnosed based on an advanced imaging such as a PET-CT scan, and the diagnosis could have been delayed in them if conventional radiological skeletal survey alone was used^18,19^. The risk of progression declined with time from diagnosis in the current cohort. The effect was most remarkable in the high-risk group. Beyond 5 years, the risk of progression in all the three groups stabilized at 3–5% per annum. A similar trend was also seen in the original Mayo Clinic study^4,5^. The rate of progression for the low-risk group was time-independent, a phenomenon seen in patients with monoclonal gammopathy of undetermined significance (MGUS). However, the yearly risk of progression in the low-risk group (5%) is much higher than the 1–2% per year rate observed in MGUS^4,30^. The pattern of progression may be due to a MGUS-like biology in the low-risk group compared to the intermediate- and high-risk groups.

We used 3-year progression as a binary end point to define optimal cutoffs for continuous variables. Patients who are at highest risk of progression do so within the first few years from diagnosis. Our cutoff of 3 years allowed us to capture these patients. The 3-year cutoff helped us to avoid missing patients who had shorter follow-up (had we taken 4 or 5 years as cutoff) or labeling patients who had progression after 2 years as non-progressors (had we taken 2 years as cutoff). BMPC% > 20%, arrived using this method was associated with a short median TTP of 29.8 months, and was a strong predictor of progression in the multivariate model; similar observations were made in the original Mayo Clinic cohort published in 2007^4^. BMPC% ≥ 20%, along with evolving changes in hemoglobin and monoclonal protein, was a strong predictor of progression in a subset of 190 patients seen at our institution between 1973 and 2014, thus lending further support to our approach^31^. We did not include evolving change in this study, as our goal was to identify patients’ risk at their first evaluation as this would be most useful for consideration in clinical trials. The changes observed during observation period allow for dynamic assessment of progression as shown by the studies examining the influence of evolving changes, and identifying additional patients at higher risk of progression^31,32^.

Restricting our analysis to only those patients who underwent an advanced imaging at diagnosis could have resulted in a cohort closer to SMM as defined by the 2014 criteria. We hypothesized that this would introduce a bias for selecting patients with higher than usual risk, considering the retrospective design of our study. We felt that patients who underwent advanced imaging at diagnosis of SMM, when it was not part of the standard-of-care investigations, could have had some other “high-risk” clinical feature, which could have alerted the treating physician, leading to an advanced imaging to exclude bone lesions. Our suspicion is partially supported by the observation that patients who had an advanced imaging tended to have shorter TTP, even though the difference was not statistically significant. Larger prospective studies are required where patients undergo an advanced imaging at diagnosis, before a definitive conclusion can be made.

On subgroup analysis in patients who had FISH testing at diagnosis, BMPC% > 20%, FLCr > 20, and high-risk cytogenetics were significant predictors of progression. This was further validated in a subset with both advanced imaging and FISH testing available. Our results suggest that with a larger cohort of patients for validation, cytogenetics will become part of a comprehensive model for risk stratification of SMM.

All patients with SMM should undergo an advanced imaging such as MRI of spine and pelvis (preferably whole body) or whole-body PET-CT scan at diagnosis to exclude the presence of focal lesions. Based on our findings in this study as well as other published data, we suggest that high-risk patients should be followed up every 2–3 months with repeat testing of hemoglobin, calcium, creatinine (and estimation of creatinine clearance), serum and urine M-protein estimation, and involved immunoglobulin level. Re-testing in intermediate-risk patients can be done at 3 months from diagnosis and then repeated every 4 months. In the low-risk group, after re-checking at 3 months, evaluation can be extended to every 6 months for 5 years, and annually thereafter. Patients who demonstrate evolution or progression in the routine tests should undergo complete evaluation including BM biopsy and skeletal imaging^19,31^.

The strengths of our study include the size of the cohort with over 400 patients, and a median follow-up of over 6 years. There are limitations in our study, related to its retrospective design, such as missing data for baseline laboratory parameters especially FISH, limited proportion of patients with advanced imaging, and lack of standardized interval assessment for progression. We defined initiation of therapy for MM in the absence of definite CRAB features as progression. Even though this may not represent progression in the strict sense, our choice reflects clinical practice, where physicians begin treatment in presence of markers which show a rapid evolution of SMM with risk of organ failure. All patients were seen at a tertiary care center specializing in PC disorders; this could be associated with a selection bias. The estimated rates of progression in our study have not been adjusted for competing causes such as death from other causes. Thus, the actual rates of progression would be slightly lower. We included patients who were treated on a clinical trial for SMM, but censored them on the date of starting treatment. The outcomes were similar in an analysis which excluded those patients. Finally, we compared the outcomes using the proposed and the conventional model in the same cohort of patients used to derive the new classification. So our results should be validated in larger independent datasets.

In conclusion, our study suggests new cutoffs for prognostic variables to risk stratify patients with SMM. BMPC% > 20%, M-protein > 2 g/dL, and FLCr > 20 are simple, routinely performed metrics, which can be used to risk stratify patients at diagnosis. Patients with two or more of these risk factors are candidates for close monitoring. The criteria can serve as a useful tool to identify high-risk patients for enrollment in clinical trials aimed at preventing and/or delaying progression. Results of cytogenetic testing should be incorporated as a prognostic factor in future models.

## Electronic supplementary material

Supplementary appendix
