# Supplementary material for: Risk stratification of smoldering multiple myeloma incorporating revised IMWG diagnostic criteria
Source: Blood Cancer J. 2018 Jun 12;8(6):59. doi: 10.1038/s41408-018-0077-4 (PMC5997745; doi:10.1038/s41408-018-0077-4)
Supplement: Supplementary file 1 — Supplementary appendix [file 41408_2018_77_MOESM1_ESM.pdf]

Comparison of the conventional Mayo Clinic model with the proposed risk stratification system using area under the curve (AUC) analysis (n=421).

2-year progression: The proposed scoring system increases the area under the curve by 9% (from 62% to 71%), which is statistically significant (p = 0.004).

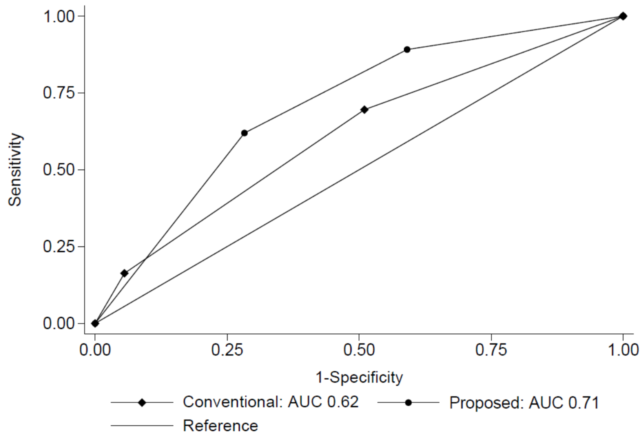

3-year progression: The proposed scoring system increases the area under the curve by 11% (from 62% to 73%), which is statistically significant (p < 0.001).

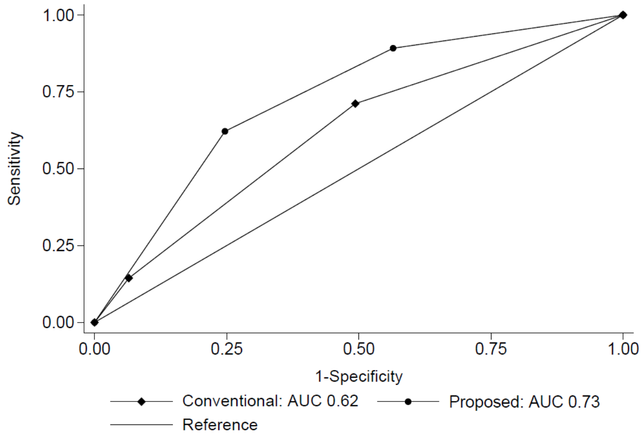

5-year progression: The proposed scoring system increases the area under the curve by 9% (from 68% to 77%), which is statistically significant (p = 0.010).

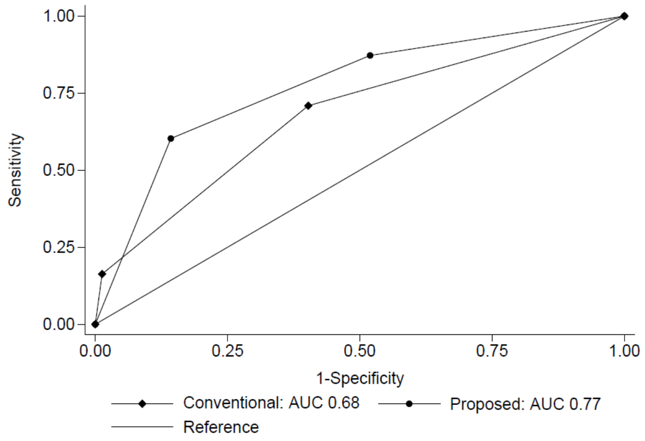

Comparison of the conventional Mayo Clinic model with the proposed risk stratification system including cytogenetics using area under the curve (AUC) analysis (n=102).

2-year progression: The proposed scoring system increases the area under the curve by 13% (from 61% to 74%), which is statistically significant (p = 0.018).

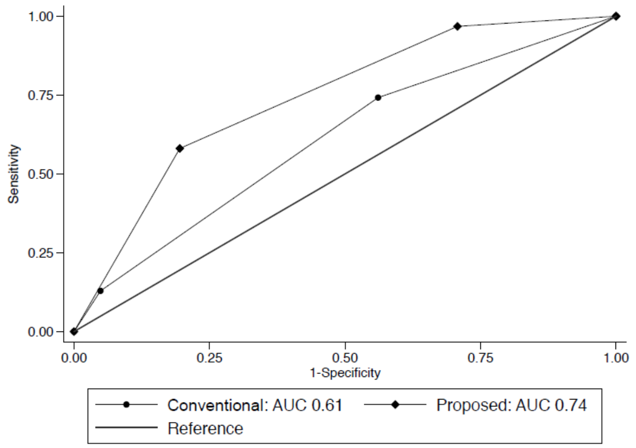

3-year progression: The proposed scoring system increases the area under the curve by 17% (from 58% to 75%), which is statistically significant (p = 0.004).

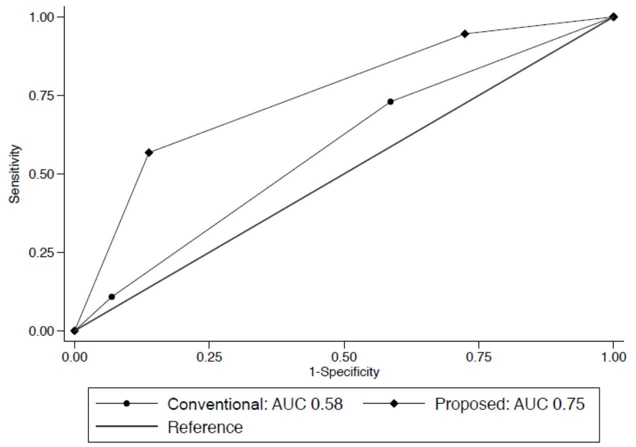

5-year progression: The proposed scoring system increases the area under the curve by 10% (from 70% to 80%), which is NOT statistically significant (p = 0.232).

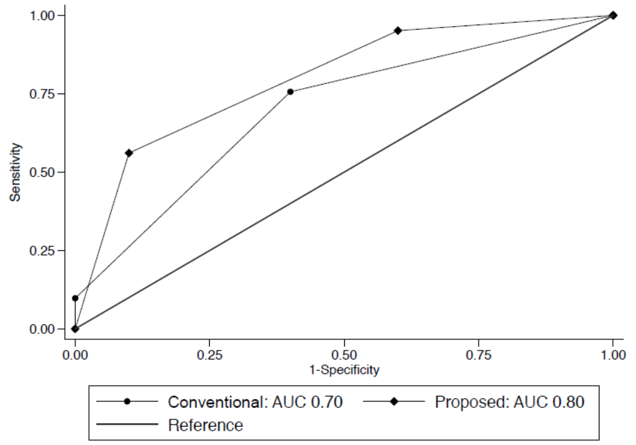

Supplementary table- Comparison of categorization of patients using the conventional Mayo Clinic model and the proposed classification system incorporating individual risk factors.

			Distribution of patients according to the conventional Mayo Clinic risk stratification system incorporating BMPC, M-protein and FLCr						
			Low-risk (n=187)			Intermediate-risk (n=193)			High-risk (n=37)
			BMPC ≥10% (n=184)	M-protein ≥3 g/dL (n=3)	FLCr ≥8 (n=1)	BMPC ≥10% + M-protein ≥3 g/dL (n=27)	BMPC ≥10% + FLCr ≥8 (n=161)	M-protein ≥3g/dL + FLCr ≥8 (n=5)	BMPC ≥10% + M-protein ≥3g/dL + FLCr ≥8 (n=37)
**Distribution of patients by the proposed risk stratification system**	Low-risk (n=143)	None (n=143)	**106**	**0**	**0**	**0**	**37**	**0**	**0**
	Intermediate-risk (n=121)	BMPC>20% (n=23)	**19**	**0**	**0**	**0**	**4**	**0**	**0**
		M-protein>2 g/dL (n=63)	**35**	**3**	**0**	**9**	**13**	**3**	**0**
		FLCr >20 (n=35)	**0**	**0**	**0**	**0**	**35**	**0**	**0**
	High-risk (n=153)	BMPC >20% + M-protein >2 g/dL (n=64)	**23**	**0**	**0**	**18**	**14**	**0**	**9**
		BMPC >20% + FLCr >20 (n=21)	**0**	**0**	**0**	**0**	**21**	**0**	**0**
		M-protein >2 g/dL + FLCr >20 (n=35)	**0**	**0**	**1**	**0**	**20**	**2**	**12**
		BMPC >20% + M-protein >2 g/dL + FLCr >20 (n=33)	**0**	**0**	**0**	**0**	**17**	**0**	**16**

BMPC indicates bone marrow plasma cell percentage; M-protein, monoclonal protein; FLCr, involved to uninvolved free light chain ratio.
